# *Trypanosoma cruzi* vaccine candidate antigens Tc24 and TSA-1 recall memory immune response associated with HLA-A and -B supertypes in Chagasic chronic patients from Mexico

**DOI:** 10.1371/journal.pntd.0006240

**Published:** 2018-01-29

**Authors:** Liliana E. Villanueva-Lizama, Julio V. Cruz-Chan, Amarú del C. Aguilar-Cetina, Luis F. Herrera-Sanchez, Jose M. Rodriguez-Perez, Miguel E. Rosado-Vallado, Maria J. Ramirez-Sierra, Jaime Ortega-Lopez, Kathryn Jones, Peter Hotez, Maria Elena Bottazzi, Eric Dumonteil

**Affiliations:** 1 Laboratorio de Parasitología, Centro de Investigaciones Regionales Dr. Hideyo Noguchi, Universidad Autónoma de Yucatán, Mérida, Yucatán, México; 2 Texas Children’s Hospital Center for Vaccine Development, Department of Pediatrics and National School of Tropical Medicine, Baylor College of Medicine, Houston, Texas, United States of America; 3 Unidad Cardiometabólica, Facultad de Medicina, Universidad Autónoma de Yucatán, Mérida, Yucatán, México; 4 Departmento de biología molecular, Instituto Nacional de Cardiología Ignacio Chávez, México D.F, México; 5 Departamento de Biotecnología y Bioingeniería, Centro de Investigación y de Estudios Avanzados del Instituto Politécnico Nacional, México D.F, México; 6 James A. Baker III Institute for Public Policy, Rice University, Houston, Texas, United States of America; 7 Department of Biology, Baylor University, Waco, Texas, United States of America; 8 Department of Tropical Medicine, Vector-Borne Infectious Disease Research Center, School of Public Health and Tropical Medicine, Tulane University, New Orleans, Louisiana, United States of America; University of Texas at El Paso, UNITED STATES

## Abstract

*Trypanosoma cruzi* antigens TSA-1 and Tc24 have shown promise as vaccine candidates in animal studies. We evaluated here the recall immune response these antigens induce in Chagasic patients, as a first step to test their immunogenicity in humans. We evaluated the *in vitro* cellular immune response after stimulation with recombinant TSA-1 (rTSA-1) or recombinant Tc24 (rTc24) in mononuclear cells of asymptomatic Chagasic chronic patients (n = 20) compared to healthy volunteers (n = 19) from Yucatan, Mexico. Proliferation assays, intracellular cytokine staining, cytometric bead arrays, and memory T cell immunophenotyping were performed by flow cytometry. Peripheral blood mononuclear cells (PBMC) from Chagasic patients showed significant proliferation after stimulation with rTc24 and presented a phenotype of T effector memory cells (CD45RA^-^CCR7^-^). These cells also produced IFN-γ and, to a lesser extent IL10, after stimulation with rTSA-1 and rTc24 proteins. Overall, both antigens recalled a broad immune response in some Chagasic patients, confirming that their immune system had been primed against these antigens during natural infection. Analysis of HLA-A and HLA-B allele diversity by PCR-sequencing indicated that HLA-A03 and HLA-B07 were the most frequent supertypes in this Mexican population. Also, there was a significant difference in the frequency of HLA-A01 and HLA-A02 supertypes between Chagasic patients and controls, while the other alleles were evenly distributed. Some aspects of the immune response, such as antigen-induced IFN-γ production by CD4^+^ and CD8^+^ T cells and CD8^+^ proliferation, showed significant association with specific HLA-A supertypes, depending on the antigen considered. In conclusion, our results confirm the ability of both TSA-1 and Tc24 recombinant proteins to recall an immune response induced by the native antigens during natural infection in at least some patients. Our data support the further development of these antigens as therapeutic vaccine against Chagas disease.

## Author summary

Chagas disease, caused by the protozoan parasite *Trypanosoma cruzi*, is a neglected tropical disease, affecting mostly marginated populations and there are no effective drugs or vaccines for prevention and/or treatment. Chronic Chagasic cardiomyopathy is the mortal form of the disease; it affects 30% of infected patients and develops after 20–30 years of infection. We propose the development of a therapeutic vaccine to prevent disease progression. Our vaccine is based on the combination of two parasite proteins: Tc24 and TSA-1. In this study we assessed the response of mononuclear cells from Mexican Chagasic patients to the stimulation by both antigens. We found that both proteins are able to recall a secondary immune response, induced during natural infection in some patients, which is characterized by memory cells that can proliferate, differentiate and produce cytokines. We also showed that the immune response is associated with some of the histocompatibility allele supertypes specific of our study population. Our results support the further development and testing of both vaccine candidate antigens in future clinical trials.

## Introduction

Chagas disease is a chronic disease caused by the protozoan parasite *Trypanosoma cruzi*, transmitted by hematophagous triatomine bugs through the direct contact of their infected feces with the skin, following a blood meal. Other transmission routes include blood transfusion, organ transplantation, congenital and oral transmission (through ingestion of food or water contaminated with infected feces) [1].

At least 5.7 million people are infected and 70.2 million are at risk of infection worldwide [2], with the majority in Latin America, but new cases of autochthonous transmission have been reported in the southern USA [3]. Moreover, due to migration patterns of infected populations, the disease has a growing presence in non-endemic regions, such as Europe or Asia [4,5]. In Mexico, the estimated prevalence is at least 0.65%, with 1.1 million people infected with *T*. *cruzi*. However, new cases of Chagas disease are underreported and the prevalence is likely much higher [6,7].

Chagas disease has two clinical phases. The acute phase presents as either asymptomatic infection or with nonspecific signs and symptoms such as fever, but parasitemia is elevated. Some acute cases (2–6%) can lead to death due to myocarditis and meningoencephalitis, mostly in children. The chronic phase is initially asymptomatic, with no clinical or physical signs of disease. However, about 20–40% of patients develop clinical symptoms after 20–30 years of infection. Chronic Chagasic cardiomyopathy is the most frequent and severe clinical manifestation. Some signs of Chagasic cardiomyopathy include abnormalities of the conduction system characterized by right bundle branch block or progressive dilated cardiomyopathy, both leading to heart failure and death. Involvement of the digestive system, such as organomegaly and gastrointestinal motor disorders, may also develop in some patients [8,9].

Control of *T*. *cruzi* infection requires the activation of both CD4^+^ Th1 and CD8^+^ T cells. Once activated, IFN-γ-producing CD4^+^Th1 cells induce activation and differentiation of CD8^+^ T cells into cytotoxic T lymphocytes, which may clear cells infected by the parasite [10–12]. However, these effector T cells die of apoptosis within a few days after activation and only a fraction of primed CD4^+^ and CD8^+^ T lymphocytes persist as antigen-specific memory T cells, which can protect against secondary challenges [13]. Albareda *et al*. 2009 showed that chronic infection promotes the exhaustion of CD4^+^ and CD8^+^ long-term memory cells, characterized by a loss of proliferation capacity and effector functions such as cytokine production. In this context, patients in early stages of chronic infection have higher frequencies of CD4^+^ and CD8^+^IFN-γ-producing cells compared to subjects with advanced disease. This suggests that induction of long-term memory cells with effector capabilities also occurs in some patients [14,15]

Benznidazole and Nifurtimox, the only two trypanocidal drugs available for Chagasic patients, can have severe side effects and limited efficacy in adults and advanced chronic patients [16,17]. A recent study indicates that Benznidazole treatment has little effect on cardiac outcomes or preventing death in patients with moderate to advanced cardiomyopathy [17]. Thus, a therapeutic vaccine aimed at preventing or at least delaying the development of chronic Chagas disease, would be an attractive and cost effective alternative or complement to current drug treatment [4,18,19].

The antigens TSA-1 (Trypomastigote surface antigen-1) and Tc24 (flagellar calcium binding protein of 24 kDa) have been proposed as candidates for an immunotherapeutic vaccine [18]. The prophylactic and immuno-therapeutic immunization with DNA vaccines encoding these antigens can decrease cardiac tissue damage and amastigote nests density in murine and canine models of *T*. *cruzi* infection [20–22]. A recombinant Tc24 protein formulated with monophosphoryl-lypid-A [23] or CpG and nanoparticles [24] can also reduce parasitemia, cardiac parasite burden and inflammatory cell infiltrate density in immunized mice compared to controls. These antigens thus appear promising for a human vaccine. Currently, a cysteine-mutagenized form of Tc24 is undergoing scale-up and production for possible clinical testing [25]. However, the extent of their recognition and processing by the human immune system and potential HLA restriction is still unclear. Most studies with Chagasic patients have focused on carriers of the A2 supertype, especially the HLA-A*0201 allele, due to its high frequency (about 45%) in Latin American populations [26,27] or with patients with unknown HLA.

In this study, we evaluated the recall immune response induced during natural infection against both rTSA-1 and rTc24 vaccine candidates using peripheral blood mononuclear cells (PBMC) from Chagasic patients and controls, as a first step towards future clinical trials of this vaccine candidate in humans. We assessed HLA diversity of our study population to understand its role in the immunogenicity of our vaccine candidates.

## Methods

### Study population

We included 20 Chagasic patients and 19 seronegative healthy controls, matched for age and gender (Table 1). Eleven of the Chagasic patients reside in the city of Merida, and the other eight patients in the rural communities of Sudzal and Teya, Yucatan. None of the Chagasic patients had received treatment before enrollment in the study. Inclusion criteria for both groups were established as follows: adults above 18 years old, born in Mexico, with parents and grandparents born in Mexico, resident in the Yucatan endemic area for at least 15 years, without history of autoimmune, immunosuppressive or infectious diseases.

**Table 1:** Demographic and clinical characteristics of the study population.

	Seronegative controls	Chagasic patients
**Number of individuals (n)**	19	20
**Gender (Female/Male)**	7/12	7/13
**Age (median (range)) (years)**	49 (30–63)	49 (31–76)
ECG principal findings*	Normal: 13/17	Normal: 12/19
	LVH: 2/17	LVH: 1/19
	IRBBB: 1/17	IRBBB: 6/19
	Bradichardy: 1/17	

LVH, Left ventricular hypertrophy; IRBBB = Incomplete right bundle branch block.

* Two individuals in the control group and one chagasic patient were not evaluated for ECG.

Diagnostic of *T*. *cruzi* infection was confirmed in Chagasic patients when at least two anti-*T*. *cruzi* antibody tests were positives, including two rapid tests (Chagas Stat-pak (CHEMBIO) and *Trypanosoma* detect (INBIOS)), and two ELISAs ((Chagatest Recombinant V.3.0 (Wiener Lab.) and an in-house ELISA based on whole parasite lysate). All seronegative controls were negative to all serological tests. An electrocardiographic recording was performed to assess clinical cardiac alterations in both groups. All participants provided written informed consent and the study was approved by the Institutional Bioethics Committee of the Regional Research Center “Dr. Hideyo Noguchi” of the Autonomous University of Yucatan (Reference #CIRB-012-0017).

### Recombinant antigens

The recombinant protein Tc24 (24.7 kDa) was expressed and purified as previously described [23]. Briefly, the Tc24 coding sequence subcloned into the yeast expression vector pPICZα was used for expression in *Pichia pastoris* X-33 and the recombinant protein was purified by affinity chromatography. For TSA-1, the coding sequence was subcloned into *E*. *coli* expression vector pET41a and purification was achieved by Ni-affinity chromatography under denaturing condition and then refolded by size exclusion chromatography. Endotoxin levels were measured with the Charles River Endosafe-PTS. The integrity and size of the recombinant proteins were analyzed by SDS-PAGE electrophoresis following purification and just before use, after storage at -80° C.

### Parasite soluble lysate

Epimastigote lysate was obtained from the H1 *T*. *cruzi* strain grown in 10% fetal bovine serum-LIT medium at 27°C. Parasites were washed twice with 1X phosphate-buffered saline (PBS), disrupted by 3 cycles of freezing (-80°C) and thawing (37°C), then sonicated in ice a total of 3 times using 45 pulses during 15 s, resting 30 s between cycles. The lysate obtained was centrifuged at 12,000 rpm during 20 min at 4°C and the soluble phase was collected. The soluble parasite lysate was assayed for protein concentration using Bradford reagent, aliquoted and stored at -80°C until used.

### ELISA to detect antigen-specific antibodies

For plasma collection, 2 mL of whole blood anticoagulated with EDTA were centrifuged at 4500 rpm during 10 min. Plasma obtained was separated, aliquoted and stored at -80°C until use. Plasmatic IgG antibodies against TSA-1 or Tc24 were measured using ELISA. Ninety six-well immunoassay plates were sensitized with carbonate buffer containing 0.2 μg/mL rTSA-1 or 1.25 μg/mL rTc24 overnight at 4°C. Plates where washed two times with wash buffer (0.05% tween 20-PBST, pH 7.4). Next, plates were blocked with 1% BSA-PBST for two hours. After washing three times with PBST, plasmas at 1:100 dilutions were added to the respective wells and incubated for 2 hours at room temperature. Plates where then washed 3 times with PBST and anti-human IgG peroxidase conjugate was added at 1:6000 dilution for incubation during 1 h at room temperature. After 3 washes with PBST, 100 μL of a substrate solution containing OPD/ citrate buffer-0.1% hydrogen peroxide was added to each well. The reaction was developed in the dark at room temperature for 30 min and stopped by adding 3N HCl. Optical densities (O.D) were read at 490 nm using a spectrophotometer. The mean O.D of each group were graphed and analyzed using Graph-PRISM software.

### PBMC isolation

Twenty mL of blood was collected from each participant by venipuncture. Approximately 16 mL of blood were collected in heparinized tubes (Vacutainer, BD) for PBMC purification and 4 mL in EDTA tubes for plasma and DNA isolation. Blood was diluted 1:1 with Dulbecco’s phosphate-buffered saline (DPBS) pH 7.4 (KCl 2.67 mM, KH_2_PO_4_ 1.47 mM, NaCl 137.93 mM, Na_2_HPO_4_ 8.06 mM) and mononuclear cell layer was separated using Ficoll histopaque-1077® (Sigma, USA) density gradient. Fresh PBS/heparinized blood was added to a Ficoll-histopaque solution at 2:1 proportion and centrifuged at 400 x g for 40 min. PBMCs were collected and washed twice with PBS pH 7.4. Cells were suspended in complete RPMI-1640 medium (RPMIc) (Gibco, USA) containing antibiotics, non-essential amino acids and 10% Fetal Bovine Serum (FBS).

### Proliferation assays

Analyses of *in vitro* proliferative response were performed using a proliferation dye (VPD-450, BD, USA). PBMCs were adjusted to 1x10^6^/mL in PBS and incubated for 10 min with 1 μM VPD-450 at 37°C. Cells were then washed and re-suspended in RPMIc adjusted to 2 x 10^5^ cells/well in 96-well plates. Cells were stimulated with 5 μg/mL Concanavalina A (Con A) (Sigma, USA), 20 μg/mL TSA-1 or Tc24 recombinant proteins or RPMI (unstimulated culture) at 37°C in 5% CO_2_. Stimulations with 20 μg/mL Bacillus of Calmette-Guérin (BCG) or 20 μg/mL *T*. *cruzi*-soluble antigen (TcSA) were used as additional controls. After 120 hours, cells were harvested, washed twice with PBS and stained for T cell phenotyping with anti-CD3-Alexa488, anti-CD4-PE and anti-CD8-PercP-Cy5.5 (all from BD, San Jose CA, USA) conjugated antibodies for 20 minutes at 4°C. Cells were fixed with 4% paraformaldehyde in PBS and 50,000 events were acquired in a FacsVerse Cytometer (BD, San Jose CA, USA), results were analyzed in FlowJo 8.7 software. A general procedure for the identification of T cell subpopulations is shown in S1 Fig. Stimulation indices were obtained by dividing the percentage of divided cells in response to antigens (experimental conditions) between the percentages of divided cells in response to RPMI (unstimulated cells). A stimulation index cut-off value of 2 was used to classify responders and non-responders [28]. For some subjects, cell counts were insufficient for running all the *in vitro* assays, and subjects with inconsistent results for methodological controls (ConA, BCG or RPMI) were removed from further analysis.

**Fig 1:**
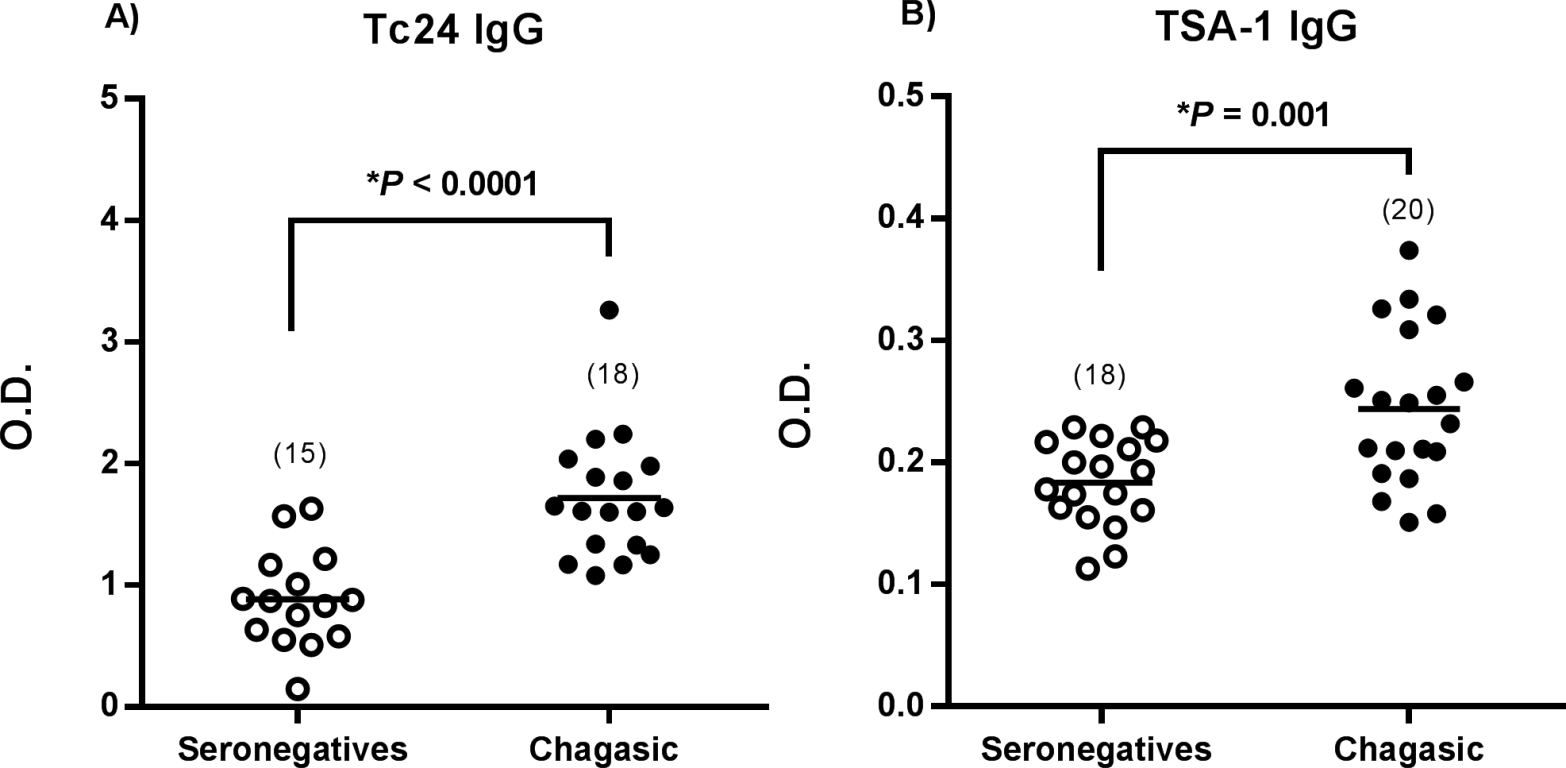
ELISA to detect antigen specific antibodies. Total IgG anti-Tc24 (**A**) and anti-TSA-1 (**B**) antibodies in seronegative controls and Chagasic chronic patients. Horizontal black lines indicate the mean optical density (O.D.) per group. *Indicates significant differences between seronegative and Chagasic patients (*P* <0.05, *t* test). Numbers in parenthesis indicate the sample size for each group.

### Intracellular cytokine staining

PBMCs were adjusted to 1 x10^6^ cells/mL in 24-well plate and stimulated with the antigens mentioned above for 22–24 hours at 37°C in 5% CO_2_. Four to six hours before the end of this incubation, 200 μL aliquots of the supernatant were collected for cytokine measurement, and 200 μL of fresh RPMIc medium with Brefeldin A (10 μg/mL) were added to block cytokine secretion. At the end of the incubation, cells were collected, washed twice with PBS and stained with anti-CD3-Alexa488, anti-CD4-PE and anti-CD8-PercP-Cy5.5 conjugated antibodies (all from BD, San Jose CA, USA). After fixation and permeabilization with Cytofix-Cytoperm (BD, San Jose CA, USA), cells were further stained with anti-INFγ-PE-Cy7 and anti-IL-10-APC (BD, San Jose CA, USA) conjugated antibodies for 20 minutes at 4°C, then fixed with 4% paraformaldehyde. Isotype-matched antibodies and basal fluorescence were assayed as controls. 100,000 events were acquired in a FACSVerse cytometer and then analyzed in FlowJo vX.0.7 (S1 Fig). The ratio of antigen-specific T cells producing cytokines was obtained by dividing the percentage of cells expressing cytokines in response to antigens (experimental conditions) by the percentage of cells expressing cytokines in response to RPMI (unstimulated cells).

### Soluble cytokines quantification

Cytokine concentrations (INF-γ, TNF, IL-10, IL-5, IL-4 and IL-2) were determined in the supernatants of stimulated PBMCs after 18–20 hours of incubation, using a cytometric bead array (CBA) kit (human Th1/Th2 kit, BD Pharmingen). Samples were processed following the manufacturer’s instructions. Briefly, 50 μL of supernatant aliquots were incubated during 3 hours with the capture beads and PE-detection reagent, then washed with wash buffer and centrifuged at 200 x g, for 5 min. Samples were acquired in a FACSVerse analyzer and cytokine concentrations were determined using BD CBA software. Samples were included when values were above the limit of detection of the cytokine: 2.4 pg/mL for IL5, 2.6 pg/mL for IL2 and IL4, 2.8 mg/mL for TNF and IL10, and 7.1 pg/mL for IFN-γ.

### Immunophenotyping of naïve and memory T cell subsets

Following antigen stimulation for 120 hours in 24-well plates as described above, PBMCs were collected, washed twice and stained with anti-CD4-V421, anti-CD45RA-FITC and anti-CCR7-PECy7 conjugated antibodies (all from BD, San Jose CA, USA) for 20 minutes at 4°C. Cells were then collected, washed twice and fixed with 4% paraformaldehyde. 100,000 events were acquired in a FACSVerse cytometer and then analyzed in FlowJo vX.0.7 to identify naïve T cells as well as effector and central memory T cells (S1 Fig). The ratio was obtained by dividing the percentage of cells expressing memory markers in response to antigens (experimental conditions) by the percentages of cells expressing memory markers in response to RPMI (unstimulated cells).

### HLA-typing

A high resolution typing was performed using SeCore HLA sequence based typing kits (Thermo Fisher Scientific) for HLA-A and HLA-B, according to the manufacturer’s instructions. Briefly, genomic DNA was extracted from 200 μL of EDTA-anticoagulated blood (For both, Chagasic patients and healthy donors), using DNAeasy blood and tissue kit (Qiagen, Hilden Germany). HLA-A and HLA-B gene fragments were amplified using primers provided by the manufacturer and purified using ExoSAP-IT Walthman, MA, USA. Sequencing was performed using Applied Biosystems BigDye Terminator purification kit and analyzed in an ABI 3130 DNA sequencer (Applied Biosystems, Foster City, CA, USA). HLA alleles were identified using UTYPE software version 6.0 (Invitrogen, Carlsbad, USA). Four digits alleles were used for identification of their respective supertype, according to the classification proposed by Sidney *et al*. 2008 [29]. Two-digit allele families were used to obtained allele frequencies for each population.

### Data analysis

The results are presented as individual point graphs, or as mean ± SEM, as indicated. Normality of the data was evaluated using the Komolgorov-Smirnov test. Parametric *t* tests or U Mann-Whitney test were used to compare the means of Chagasic patients and healthy donors for proliferation assays, immunophenotyping of memory T cells, ICS and ELISA assays. Contingency tables for responders and non-responders per group were build for proliferation assays, immunophenotyping and ICS, and comparison of proportion data between groups were analyzed by Chi square or Fisher. Graphs were built and statistics obtained by using GraphPad-Prism 7 software. Differences were considered significant for *P* values < 0.05.

HLA supertype frequencies were compared by Fisher test and odds ratios were calculated with their 95% confidence interval. A network of the various components of the immune response following antigen stimulation was constructed in Cytoscape 3.5.0 [30] to visualize the overall immune response. Circular nodes represent the major immune parameters tested and the edges connecting the parameters show significant differences between Chagasic patients and controls.

## Results

### Study population

A total of 20 patients were confirmed as positive for *T*. *cruzi* antibodies using serological commercial tests and our in-house ELISA against total soluble *T*. *cruzi* antigens. They lived in the city of Merida or in rural Yucatan and had been diagnosed with *T*. *cruzi* infection for the first time in 2004–2006. They were between 31 and 76 years old. Age-matched seronegative healthy donors (n = 19) were from the same region and ascendency.

EKG recording indicated that most patients and controls had normal EKG (no arrhythmias or other conduction disorders) (Table 1), and minor alterations such as incomplete right bundle branch block (IRBBB) were found similarly in patients and controls (F = 7.38, d.f = 4, *P* = 0.09). These electrocardiographic alterations were not specific for Chagas disease and Chagasic patients could thus be classified in the asymptomatic chronic phase of Chagas disease.

### Naturally induced TSA-1 and Tc24 specific IgG antibodies

We evaluated the specific humoral immune response against Tc24 and TSA-1 antigens triggered by natural infection. We observed significantly higher anti-Tc24 (Fig 1A, P<0.0001, *t* test) and anti-TSA-1 (Fig 1B, *P* = 0.001, *t* test) IgG antibody levels in plasma from Chagasic patients compared to the seronegative controls. This confirmed that antibodies induced during natural infection recognized both recombinant antigens Tc24 and TSA-1 and suggested the presence of B cell-responses specific for these proteins in Chagasic patients.

### TSA-1 and Tc24 antigens can recall memory T cells induced by natural infection

To evaluate the presence of Tc24 and TSA-1 antigen-specific memory T cells induced upon natural infection in Chagasic patients, we first measured the proliferative recall response. Proliferation assays were carried out by stimulating PBMCs from Chagasic patients and healthy donors with rTSA-1 or rTc24 antigens. BCG vaccine, *T*. *cruzi* SA, Concanavalin A (ConA) and RPMI were used as controls. ConA-stimulated cells from all subjects presented a very high stimulation index (range of 50–120, S2A Fig). Similarly, methodological controls using cell stimulation with BCG and TcSA showed antigen-specific T cell proliferation in BCG-vaccinated and Chagasic patients, respectively (S2B and S2C Fig).

**Fig 2:**
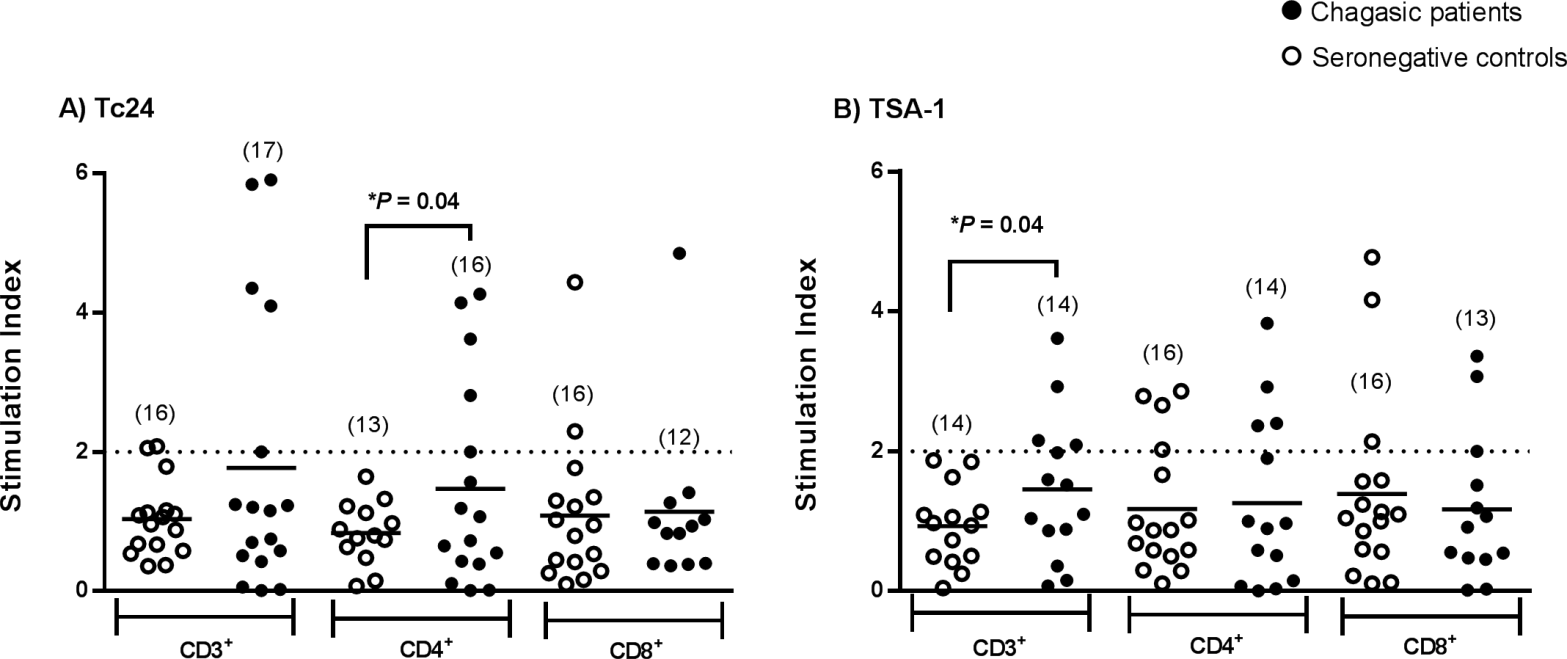
Antigen-specific proliferation assay. Stimulation index of CD3^+^, CD3^+^CD4^+^ and CD3^+^CD8^+^ T cells populations from seronegative controls (open circles) and Chagasic patients (Black circles), after stimulation with rTc24 (**A**) or rTSA-1 (**B**). Horizontal black lines indicate the mean. The dotted horizontal line shows the cut-off value to identify responders/non-responders. *Indicate significant differences in the proportion of responders between seronegative and Chagasic patients (*P* <0.05, Fisher test). Numbers in parenthesis indicate the sample size for each group.

Proliferation assays with our recombinant proteins showed that after stimulation with rTc24, CD3^+^ and CD4^+^ T cells from Chagasic patients tended to have a higher stimulation index compared to the seronegative group. In addition, we found a significantly higher proportion of Chagasic patients with CD4^+^ T cells that proliferated after stimulation with rTc24 compared with controls (Fig 2A; *P* = 0.04, Fisher test). Similarly, more Chagasic patients had CD3^+^ T lymphocytes that proliferated (stimulation index ≥2) after stimulation with rTSA-1 compared to the control group (Fig 2B; *P* = 0.04, Fisher test). On the other hand, we found no differences in the CD8^+^ T cell population recalled by Tc24 or TSA-1. Together, these results indicated presence of Tc24 and TSA-1-specific memory cells induced during natural infection in Mexican Chagasic patients.

### Tc24 antigen-specific CD4^+^ memory cells of Chagasic patients have a T_EM_ phenotype (CD45RA^-^CCR7^-^)

**Fig 3:**
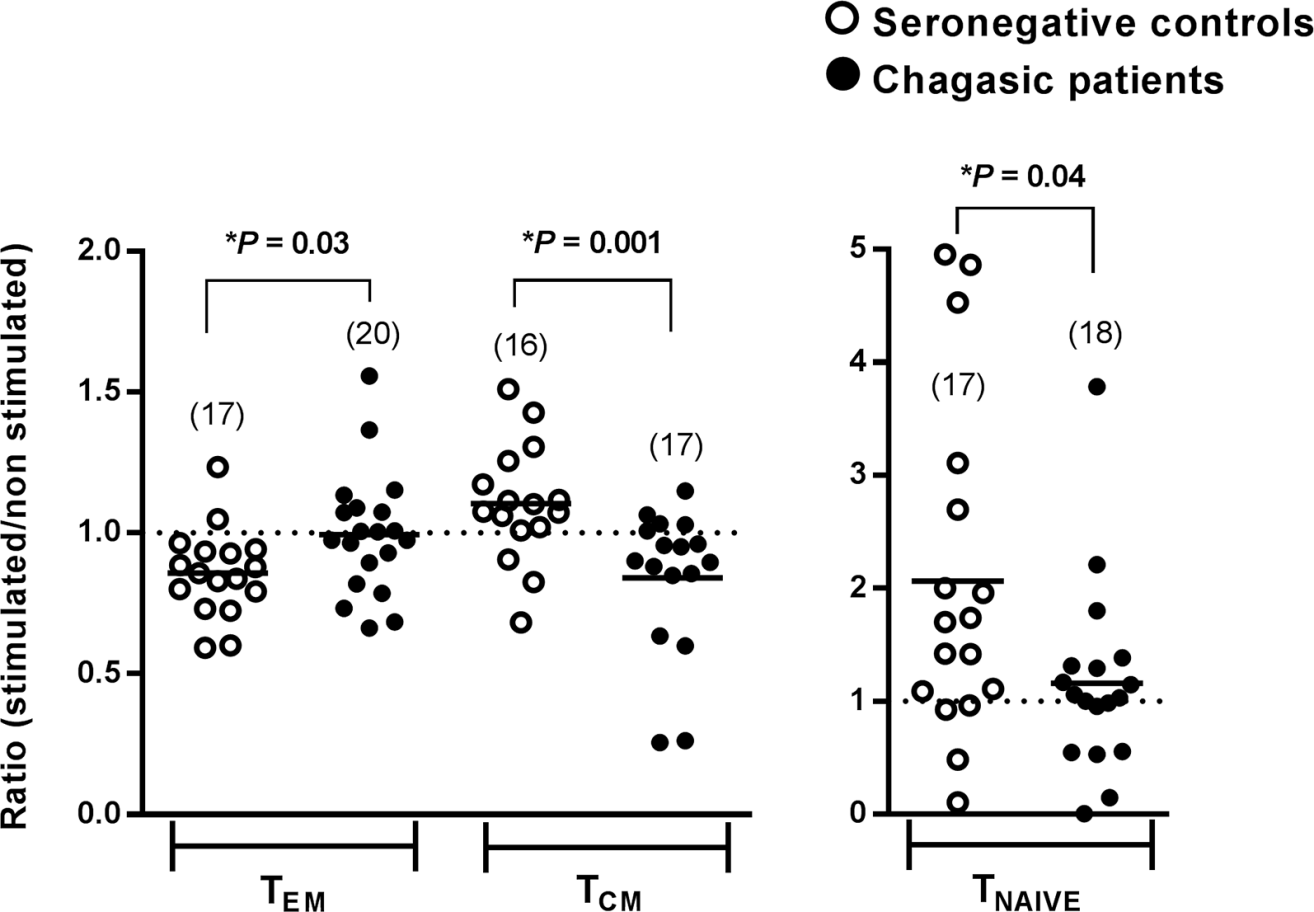
Phenotyping of CD4^+^ T memory cells. PBMC cells from seronegative controls (open circles) and Chagasic patients (black circles) were stimulated with rTc24, and phenotyped as T effector memory (T_EM_), T central memory (T_CM_) or T naïve (T_NAIVE_) cells. Horizontal black lines indicate the mean of the group. *Indicates significant differences between seronegative and Chagasic patients (P <0.05, *t* test for parametric distribution or U Mann-Whitney for non-parametric distribution). Numbers in parenthesis indicate the sample size for each group.

We then characterized the phenotype of these memory cells using CD45RA and CCR7 as memory markers, as previously described [31,32]. Cell stimulation with ConA induced a strong response in both controls and Chagasic patients (S3A Fig), and stimulation with TcSA induced a trend to a higher ratio of T_CM_ memory cells in seropositive patients when compared to the seronegative group (S3B Fig). On the other hand, when using rTc24 the results showed a significantly higher ratio (P = 0.03, *t* test) of Tc24-specific CD4+ T_EM_ cells, as well as a significantly lower ratio of Tc24-specific T_CM_ (P = 0.003, U Mann-Whitney test) and T_NAIVE_ cells (P = 0.03, U-Mann-Whitney test) in Chagasic patients compared to seronegative individuals (Fig 3). We also found a significant lower ratio (P <0.05) on naïve CD4+ T cell population (T_NAIVE_) of seropositive patients compared to seronegative group, after stimulation with TSA1 (S4 Fig).

### PBMC from Chagasic patients present antigen-specific cytokine production

**Fig 4:**
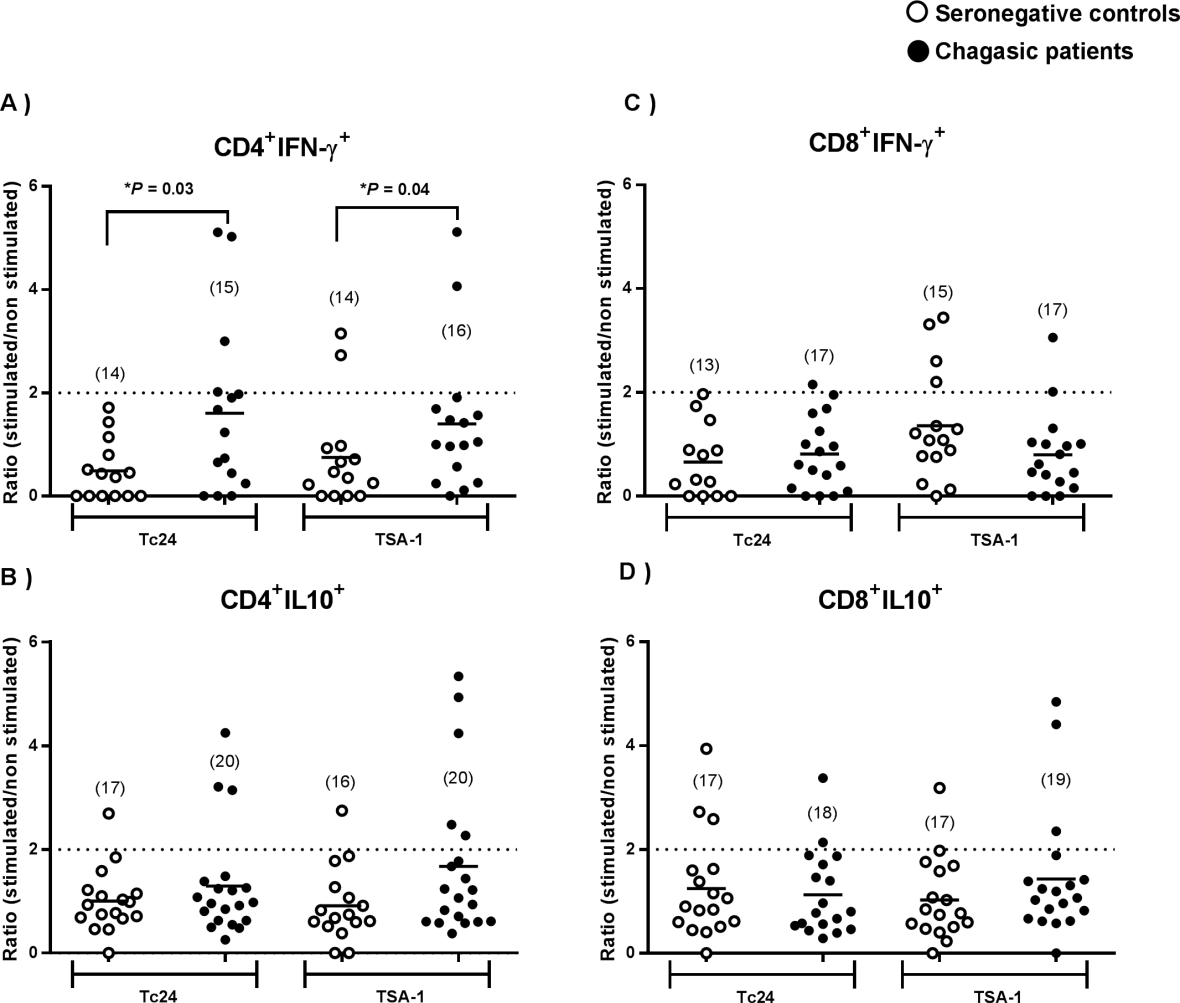
Antigen-specific cytokines produced by CD4^+^ and CD8^+^ T cells. Stimulation index of CD4^+^ (**A, B)** and CD8^+^ (**C, D)** T cells expressing INF-γ or IL-10 in a group of *T*. *cruzi* seronegative controls (open circles) and Chagasic patients (black circles). Horizontal lines indicate the mean. *Indicate significant differences between seronegative and Chagasic patients (P<0.05, U Mann-Withney). Numbers in parenthesis indicate the sample size for each group.

We then analyzed the T cell cytokine production in response to antigen stimulation using two approaches: an intracellular cytokine staining assay to phenotype INF-γ and IL10 producing T cells, and a CBA to measure Th1 and Th2 secreted cytokines (INF-γ, TNF, IL10, IL5, IL4 and IL2). Again, stimulation with ConA resulted in a strong response in both groups of subjects (S5A Fig) and stimulation with TcSA induced some antigen-specific cytokine production in Chagasic patients (S5B Fig). Intracellular cytokine staining also showed that PBMC from Chagasic patients had a significantly higher ratio of CD4^+^ T cells producing INF-γ after stimulation with both TSA1 and Tc24 (Fig 4A), when compared to the control group (P = 0.03 and P = 0.04, respectively, U Mann-Withney). The ratio of IL-10-producing CD4^+^ T cells tended to be higher in Chagasic patients, but this did not reach statistical significance (Fig 4B). Also, cytokines produced from CD8^+^ T cells after stimulation with TSA1 or Tc24 were comparable between both groups (Fig 4C and 4D).

**Fig 5:**
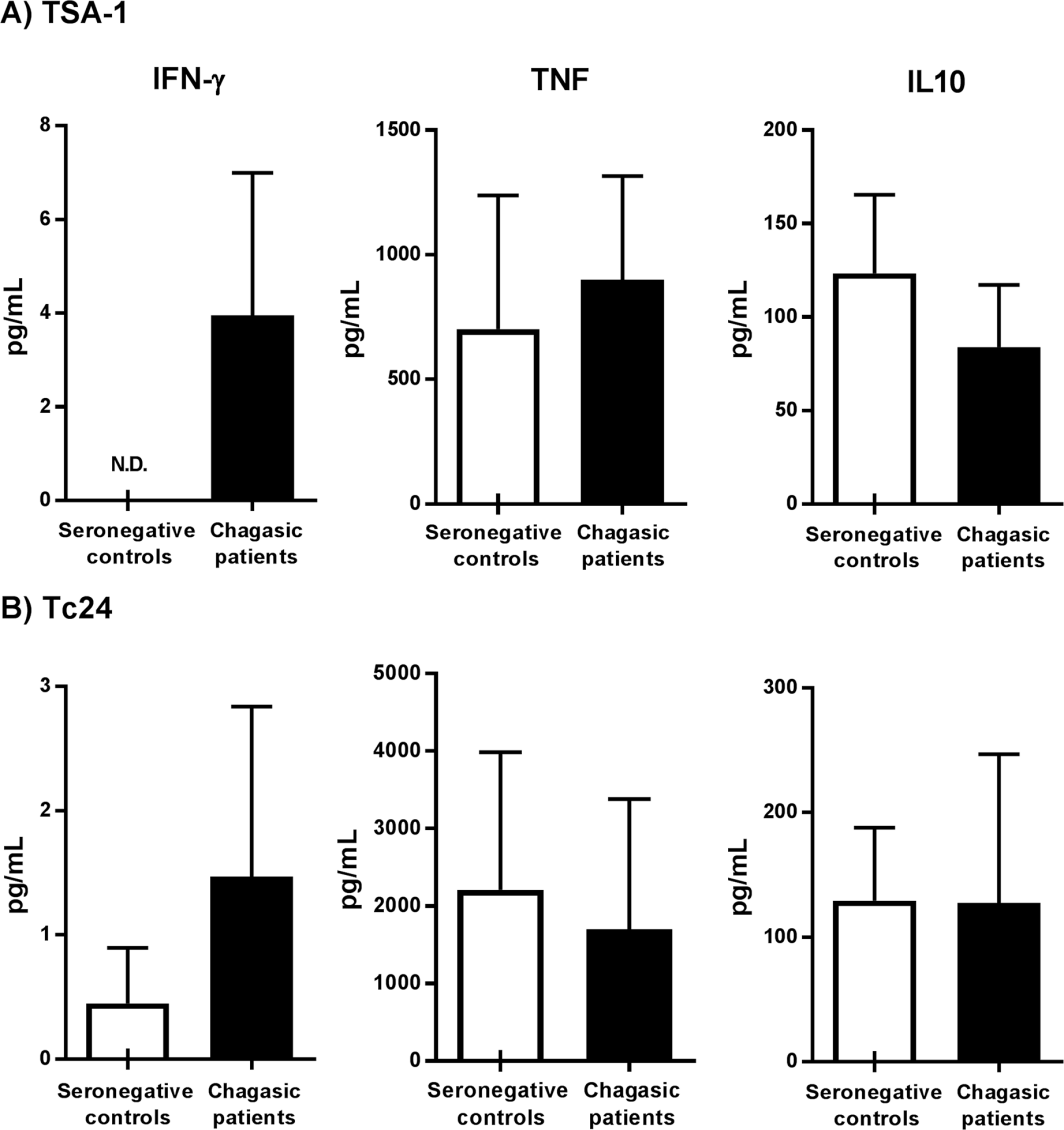
Quantification of Th1 and Th2 soluble cytokines. Th1 and Th2 cytokines were measured in supernatant of cultures after stimulation with TSA-1 (**A**) or Tc24 (**B**). Graphs show the concentration in pg/mL comparing seronegative control and Chagasic patients for each cytokine. N.D.: Not detected. Results include 13 Chagasic patients and 7 seronegative controls.

The profile of cytokine secretion of PBMC, measured by CBA, in response to rTSA-1 (Fig 5A) or rTc24 (Fig 5B) confirmed a high INF-γ secretion by cells from Chagasic patients compared to the control group. Pro-inflammatory TNF was the cytokine secreted with the highest levels in the supernatant of PBMC after stimulation, however no significant differences were observed between groups, neither for IL10 secretion levels. On the other hand, IL2, IL4 and IL6 were not detected in response to rTc24 or TSA-1 in both groups.

### Immune response network recalled by TSA-1 and Tc24 antigens

**Fig 6:**
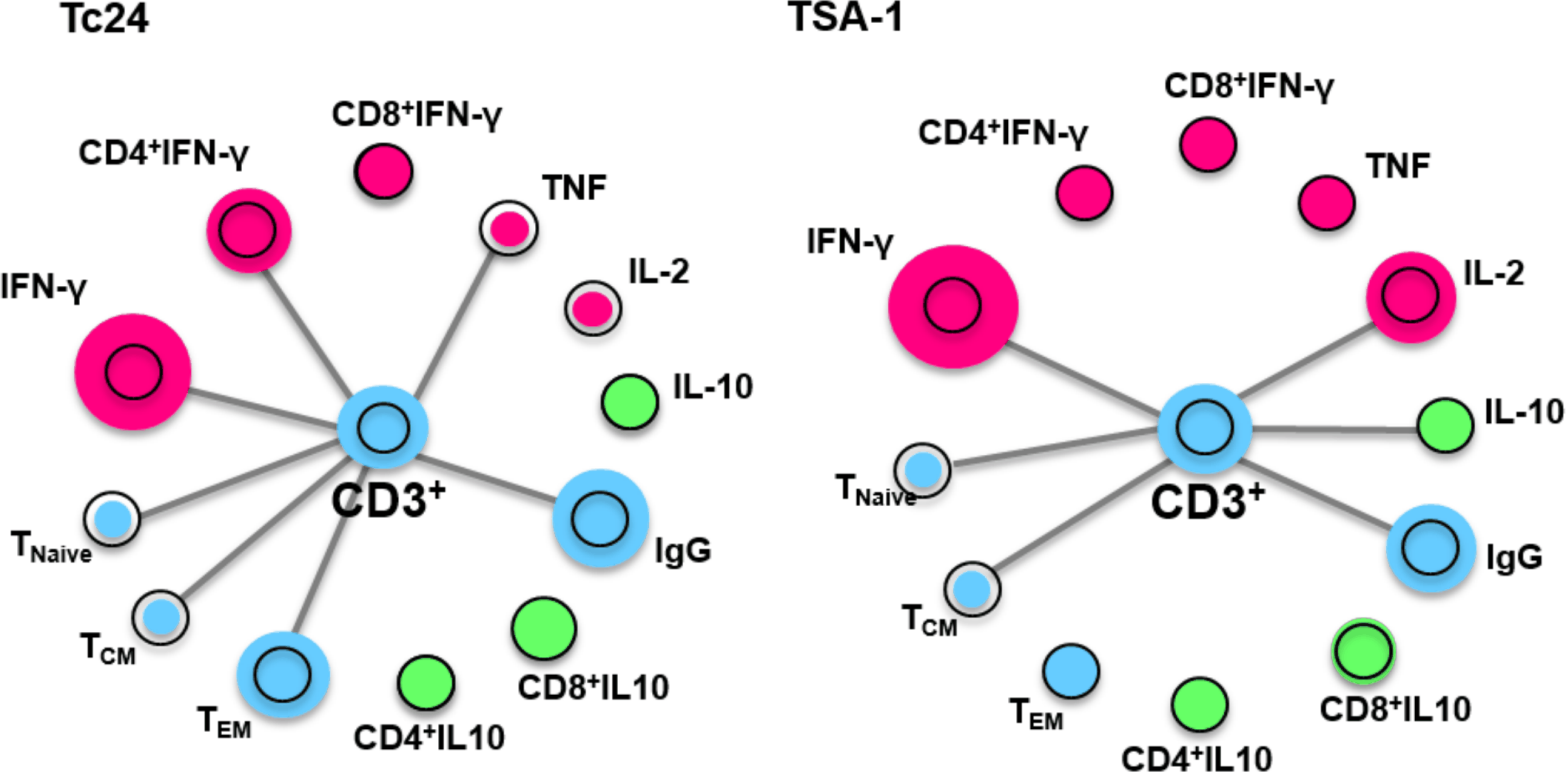
Antigen-specific immune network. Networks were constructed using Cytoscape to provide an integrated view of Tc24 and TSA-1 recall responses in Chagasic patients and seronegative controls. Circle nodes represent T cell populations and cytokines, with the black circle corresponding to seronegative controls, and the colored circles corresponding to the seropositive Chagasic patients. A larger size of the circle indicates activation, while a smaller size indicates an inhibition/reduction compared to the seronegative controls. Pink colors are associated with a pro-inflammatory Th1 profile, while green is associated with an anti-inflammatory immune profile. Blue reflects non-polarized immune responses. Edges link nodes showing differences between Chagasic patients and controls.

To visualize the different immune parameters induced by TSA-1 and Tc24, we constructed a network with nodes representing the various immune components measured above. Edges link the parameters showing differences between Chagasic patients and seronegative controls, which are also indicated by the size of the nodes. As shown in Fig 6, Both Tc24 and TSA-1 antigens activated several T cell populations, and the cytokine profile was predominantly biased towards IFN-γ and a Th1 pro-inflammatory profile. Indeed, Tc24 induced a shift from naïve and central memory cells to effector memory T cells, together with the activation of IFN-γ production, particularly by CD4^+^ cells. However, IL2 and TNF production tended to decrease. TSA-1 stimulation induced both IFN-γ and IL-2 production but led to decreases in IL10, naive and central memory T cell populations, suggesting an immune response biased to Th1.

### HLA typing and association with immune response

To understand the role of HLA allele diversity in the immunogenicity of our vaccine candidates we first identified the HLA repertoire of our study population. The HLA-A and B genotypes of Chagasic patients and healthy donors were determined using high resolution typing by sequencing. For HLA-A, eight allele families were identified in Chagasic patients compared to 12 allele families in the control group, corresponding to at least 4 A-supertypes. For HLA-B, there were 9 alleles families in the Chagasic patients compared to 12 in the control group including 3 supertypes in Chagasic patients and 4 different supertypes in the group control (S1 Table).

The most frequent HLA-A allele supertype in our population was A03 (33/71, 46.5%), followed by A24 (17/71, 23.9%), A02 (11/71, 15.5%) and A01 (10/71, 14.1%). For HLA-B allele supertypes, the most frequent allele was B07 (25/52, 35.2%, followed by B27 (14/52, 19.7%), B44 (11/52, 15.5%), and B62 (2/52, 2.8%). The predominance of A03 supertype observed in our population is markedly different from other indigenous and mestizo populations in Mexico, in which A02 alleles predominate, highlighting the unique characteristics and genetic origin of the Yucatan population (S2 Table).

While most HLA-A and B supertypes were distributed evenly between controls and Chagasic patients, there were significant differences for HLA-A01 and A02 (Table 2). Indeed, A01 was significantly less frequent, while A02 was significantly more frequent in patients compared to controls. HLA-B44 also tended to be less frequent in patients, but this did not reach statistical significance (Table 2). These results suggested potential association of these HLA alleles with *T*. *cruzi* infection in this population.

**Table 2:** Frequency of HLA-A and HLA-B supertypes in the study population.

	Phenotype			Genotype		
	Patients	Controls	Odds Ratio	Patients	Controls	Odds Ratio
ST	Frequencyn = 19 (%)	Frequency n = 19 (%)	OR (95% CI), *P* value	Frequency2n = 38 (%)	Frequency 2n = 38 (%)	OR (95% CI), *P* value
A01	1 (3.1%)*	**7 (22.6%)**	**0.09 (0.01–0.81) *P* = 0.02**	1 (2.6%)*	**9 (23.7%)**	**0.09 (0.01–0.73), *P* = 0.01**
A02	9 (28.1%)*	**3 (9.7%)**	**4.80 (1.04–22.10) *P* = 0.04**	10 (26.3)*	**3 (7.9%)**	**4.17 (1.04–16.61), *P* = 0.03**
A03	14 (43.7%)	12 (38.7%)	1.6 (0.41–6.51) *P* = 0.36	17 (44.7%)	16 (42.1%)	1.11 (0.45–2.76), *P* = 0.50
A24	8 (25.0%)	5 (16.1%)	2.04 (0.52–7.99) *P* = 0.24	10 (26.3%)	6 (15.8%)	1.90 (0.61–5.91), *P* = 0.20
A other	0 (0.0%)	4 (12.9%)	0, *P* = 0.053	0 (0.0%)	4 (10.5%)	0, P = 0.11
ST	Frequency n = 17 (%)	Frequency n = 16 (%)	OR (95% CI), *P* value	Frequency 2n = 34 (%)	Frequency 2n = 32 (%)	OR (95% CI), *P* value
B07	11 (68.8%)	11 (64.7%)	0.83 (0.19–3.56), *P* = 0.53	13 (38.2%)	12 (37.5%)	1.01 (0.38–2.79), *P* = 0.57
B27	6 (35.3%)	6 (37.5%)	0.91 (0.22–3.76), P = 0.59	8 (23.5%)	6 (18.7%)	1.33 (0.40–4.38, *P* = 0.43
B44	3 (17.6%)	8 (50.0%)	0.21 (0.04–1.05), *P* = 0.07	4 (11.8%)	8 (25.0%)	0.40 (0.11–1.49) *P* = 0.14
B62	0 (0.0%)	2 (12.5%)	0, P = 0.23	0 (0.0%)	2 (6.3%)	0, P = 0.23
B other	6 (35.3%)	3 (18.5%)	2.36 (0.47–11.72), *P* = 0.25	5 (14.7%)	4 (12.5%)	1.21 (0.29–4.96), *P* = 0.54

ST: Supertypes; OR: Odds ratio; CI: Confidence interval.

*Indicates a significant difference in frequency between patients and controls (Fisher test).

**Fig 7:**
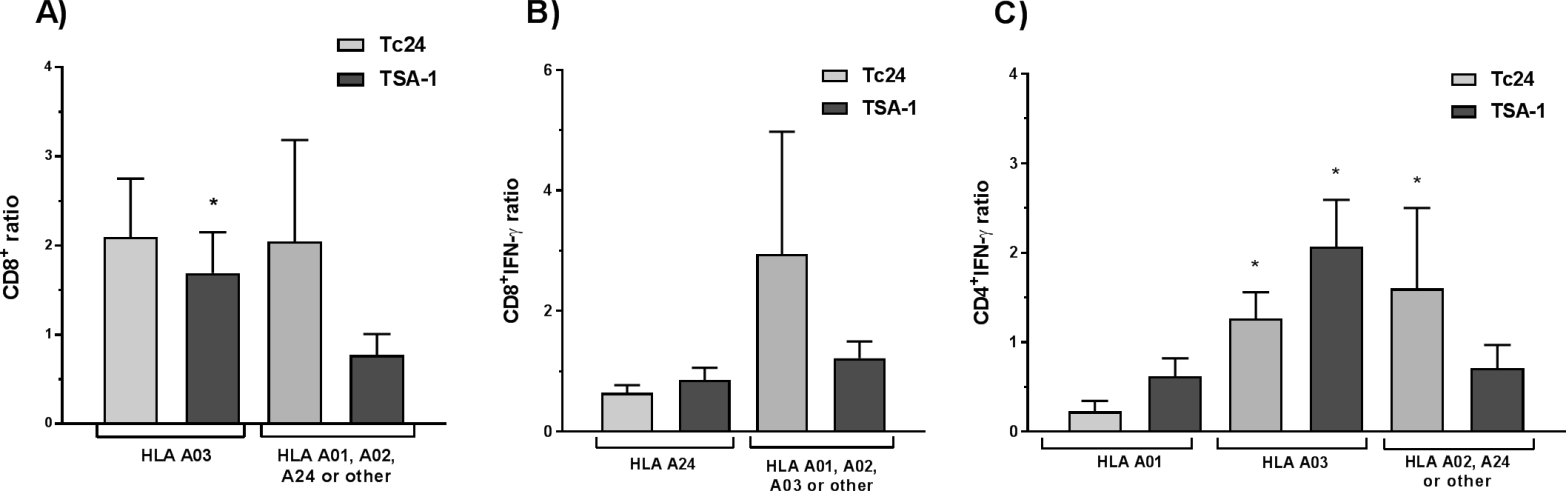
HLA restriction of antigen-specific immune response. The antigen-specific immune responses were analyzed according to the HLA supertypes of the study participants. Data are presented as mean ± SEM for the induction of CD8^+^ T cell proliferation (**A**), CD8^+^-IFN- γ ratio (**B**), and CD4^+^ IFN-γ ratio (**C**). *Indicates a significant difference in the antigen-specific immune response between HLA supertypes.

To evaluate the relationship between the HLA allele supertypes of the study participants and the variables of the immune response against TSA-1 and Tc24, we tested for potential differences in immune response based on the HLA supertype. Interestingly, we did not detect significant HLA restriction for most of the immune parameters that were evaluated, with the notable exceptions of IFN-γ production by CD4^+^ and CD8^+^ T cells, as well as CD8^+^ T cell proliferation. Indeed, for TSA-1 but not for Tc24, CD8^+^ proliferation was restricted to HLA-A03 (Fig 7A); for Tc24 but not for TSA-1, the CD8^+^IFN-γ response was restricted to HLA-A01, A02 or A03 compared to A24 (Fig 7B), while for TSA-1 and Tc24, HLA-A01 was associated with a low CD4^+^IFN-γ response, compared to HLA-A02, A03 or A24 alleles (Fig 7C). In spite of these HLA restrictions, and based on their allele frequency of our study population, as well as in other Mexican populations (S2 Table), we can expect to have at least a partial response to either of the candidate antigens in a majority of individuals (28–70%).

## Discussion

Based on the promising results obtained with TSA-1 and Tc24 antigens as vaccine candidates in pre-clinical models [23,24,33,34], we investigated here the ability of the TSA-1 and Tc24 recombinant proteins to recall a specific immune response in PBMC of Chagasic patients, as a first step towards clinical trials. Overall, our measurements of specific antibodies, T cell proliferation, memory cell phenotyping and cytokine production confirm the recall response induced by both Tc24 and TSA-1 *T*. *cruzi* recombinant antigens in at least some naturally infected patients and therefore, support their future development as therapeutic vaccines.

The higher proliferation response induced by rTSA1 and rTc24 in CD3^+^ and CD4^+^ T cells of seropositive patients suggested the presence of antigen-specific memory cells induced by natural *T*. *cruzi* infection in these Mexican patients. Proliferation response to recombinant proteins has been previously reported [35,36] suggesting a protective role to *T*. *cruzi* infection. Moreover, using *T*. *cruzi* soluble antigen, we also found *T*. *cruzi*-specific proliferative response in the T cell compartment (CD3^+^, CD4^+^ and CD8^+^) of seropositive subjects, whose response was higher when was compared to seronegative individuals, as has been previously reported (S3 Fig). Such proliferative response is also remarkable in the context of immune exhaustion in chronic Chagas disease. Indeed, it has been demonstrated that CD4^+^ and CD8^+^ T cells of chronic Chagasic patients have lower proliferation ability compared to patients in the indeterminate phase [15,37–39]. This is in agreement with the classification of our cohort of patients in the indeterminate stage based on the very mild cardiac alterations that they presented.

Nonetheless, not all Chagasic patients responded to the antigens, which may be due their HLA supertypes (See below) and/or to potencial limitations of our experimental approach, highlighting the complexity of studying immune responses in patients compared with mouse models.

Next, we characterized the phenotype of memory cells that proliferated in response to stimulation with our candidate proteins. Peripheral blood T cells can be divided into naïve and memory cells. This division is based on their functions and cell surface markers. Together, expression of the CD45RA^+^ isoform and the chemokine receptor CCR7 permit further discrimination of central memory T cells (CD45RA^-^CCR7^+^) and effector memory T cells (CD45RA^-^CCR7^-^) [31,32]. Fiuza *et al*. (2009) have described the memory profile of peripheral CD4^+^ and CD8^+^ T lymphocytes as well as its cytokine secretion, before and after *in vitro* antigenic stimulation (using total soluble antigen of *T*. *cruzi*) between the different clinical forms of Chagas disease. They found that Chagasic patients (in indeterminate and cardiac stages) have lower percentages of naïve CD4^+^ and CD8^+^ T cells as well as higher percentages of memory CD4^+^ T cells in infected individuals. They also observed that individuals in the indeterminate phase presented more T_CM_ CD4^+^ T cells, and suggested that it may induce a regulatory mechanism to protect the host against the exacerbated inflammatory response elicited by the infection [40]. However, these results were obtained by recalling the whole memory cell compartment with total soluble antigens. In our study, we also observed a higher frequency of T_CM_ memory cells after ASTc stimulation in seropositive patients when compared to seronegative group (S3 Fig), as previously reported in Brazilian chronic Chagasic patients [40]. The central memory T cell (T_CM_) subpopulation has a higher sensitivity to antigen stimulation, is less dependent on co-stimulation and can work as CPA to dendritic and B cells. The process of differentiation of memory T cells subsets is still not clearly understood, but it is generally accepted that naïve cells antigenic activation can originate T_CM_ and these later differentiate into T_EM_ cells [31,40].

After stimulating PBMCs with our protein antigens, we observed a higher ratio of Tc24-specific CD4^+^ T_EM_ cells, as well as a lower ratio of Tc24-specific T_CM_ cells in Chagasic patients compared to seronegative controls. This finding can represent a switch in memory T cell compartment in response to stimulation with TSA-1 and Tc24 recombinant proteins, with activation of T_CM_ memory T cells generating T_EM_ cells. Functionally, T_EM_ have a higher capacity for cytokine production and less proliferative response than T_CM_ and T_NAIVE_ [31]. On the other hand, the naïve T cell population was expected to have the same frequency between experimental groups. The lower ratio of naïve T cells found in Chagasic patients could be due to a lower basal frequency of CD4^+^ naïve T cells, as has been previously reported in chronic Chagasic patients. This lower frequency of naïve cells could also be explained by the to continuous re-stimulation of this cell compartment during the chronic infection.

The profile of memory cells against recombinant proteins has been poorly studied in humans; most of the findings were made using total soluble *T*. *cruzi* antigens or focused on CD8^+^ populations. Albareda *et al* 2006, showed that the percentage of CD8^+^CD45RA^-^CCR7^-^ (T_EM_) T cells in individuals with indeterminate Chagas disease was significantly higher than in the uninfected group after co-culture with *T*. *cruzi* infected autologous dendritic cells [14]. In contrast, the percentage of central memory cells (CD8^+^CD45RA^-^CCR7^+^) was similar to the controls, suggesting that subjects in the early stages of disease have memory T cells capable of rapid effector functions [14]. We observed a similar phenomenon in CD4^+^ cells after stimulation with rTc24, which could mediate protection against the development of cardiac pathology. Recently, Egui *et al* (2015) reported a memory T phenotype specific of the TcCA-2 protein, characterized by CD8^+^ T_NAIVE_ cells in patients in asymptomatic stage and mainly CD8^+^ effector memory cells (T_EM_ and T_EMRA_) in chronic cardiac patients [41]. However, predominance of T_NAIVE_ cells in CD8^+^ cells specific of the TcCA-2 protein suggests a low antigenicity, at least during the early stages of the disease. We did not evaluate the CD8^+^ cells memory compartment here, but future studies could focus on CD8^+^ T cells specific of TSA1 and Tc24 recombinant proteins.

Characterization of the cytokine profile in response to stimulation with TSA-1 or Tc24 further confirmed their activation of the immune response. It is known that IFN-γ and IL10 cytokines have antagonist functions due to the pro-inflammatory role of IFN-γ, while IL10 can mediate regulatory or anti-inflammatory functions. CD4^+^ T cells of Chagasic patients trended to express high amounts of both IFN-γ and IL10 in response to TcSA stimulation, relative to seronegative subjects (S5 Fig). Additionally, we found that TcSA tended to induce somewhat higher IFN-γ-specific CD4^+^ T cells compared to IL10-producing CD4^+^ cells in Chagasic patients. This finding was consistent with a previous study, which suggested that chronic Chagasic patients have more CD4^+^ memory cells producing both IFN-γ and IL10 cytokines [40]. In our study, after stimulation with TSA-1 or Tc24, CD4^+^ T cells of seropositive subjects had a higher IFN-γ production. Moreover, the number of patients showing IFN-γ in response to Tc24 stimulation was significantly higher in Chagasic patients compared with seronegative controls. This suggests that Tc24 protein tended to recall a Th1 immune response in most of the patients. However, no differences were found in either IFN-γ or IL10 production in CD8^+^ T cells. It is known that *in vitro* activation of CD8^+^ cells requires specialized antigen-presenting cells, which may not have been present in sufficient proportion in our culture conditions.

In addition, measurement of secreted cytokines following stimulation with rTSA-1 or rTc24 showed enhanced levels of IFN-γ in Chagasic patients, compared to seronegative controls. This finding confirmed the ability of our vaccine candidate proteins to induce cytokine production. TNF, another pro-inflammatory Th1 cytokine, had the highest concentration detected by CBA (1500–5000 pg/mL) in comparison with IFN-γ (3–8 pg/mL) and IL10 (200–300 mg/mL), however no differences were found when comparing TNF levels between groups (patients and controls). Overall, the profile of secreted cytokines in response to TSA-1 and Tc24 showed no clear polarization for TSA-1 and a bias towards Th1 for Tc24.

The role of HLA diversity was also tested as one of the factors that can influence immunogenicity of antigens. Interestingly, HLA diversity of our study population was markedly different from that of other indigenous or mestizo populations in Mexico, highlighting the uniqueness of the Yucatan population (S2 Table). The most frequent alleles observed corresponded to A03 and B07 supertypes, while most studies conducted on human Chagas disease have focused on patients with the A02 allele family, which is of high frequency in Latin American populations. Thus, cytotoxic T lymphocytes specific for peptides derived from TSA-1, ASP-1 and ASP-2 have been detected in Chagasic patients with HLA-A02 from Guatemala [42].

Additional CD8^+^ epitopes restricted to A02 alleles and derived from different antigens including cruzipain, FL-160, KMP-11, HSP-70, PFR1-4, and other trans-sialidases proteins have been identified in HLA-A*0201 Chagasic patients [26,27,41,43–46]. Our findings suggest the necessity to include a greater diversity of HLA supertypes in Chagas disease studies. For example, Alvarez and collaborators (2008) screened transialidase proteins from the *T*. *cruzi* genome against six commons HLA-I supertypes and identified that peptides predicted to bind the A02 supertype were most frequently recognized in Chagasic patients (regardless of their HLA), followed by peptides binding to the A03 and A24 supertypes [44]. Thus, vaccine candidates should be tested in patients with a variety of HLA profiles, as shown by Lasso *et al*. (2016), who reported an epitope of KMP-11 protein that can be presented in the context of more than one HLA-I supertype (A02, A24 and A01) in Colombian Chagasic patients [27].

While HLA allele frequencies were mostly similar between Chagasic patients and seronegative controls, we did detect some significant differences, suggesting that HLA-A 01 and A02 supertypes could be associated with protection and susceptibility to *T*. *cruzi* infection, respectively. Most studies have focused on the role of HLA class II alleles in *T*. *cruzi* infection and/or disease progression, with HLA-DQ1 and DQ7 [47], DRB1 [48–52] and DR4 [53] being found associated with susceptibility or resistance to infection/disease progression in different populations. However, Class I HLA, such as HLA-C*03, was associated with susceptibility to the development of Chagasic cardiomyopathy in Venezuelan patients [54], HLA-B*39 was found to be more frequent in Mexican Chagasic patients compared to healthy controls and A*68 and B*35 (allele families) were associated with disease progression [53].

As expected, we detected some HLA associations with the immune response recalled by TSA1 and Tc24 antigens, particularly for CD8^+^ T cell proliferation and IFN-γ production. The association of HLA-class I alleles with CD4^+^ responses was not expected, but may be due to the main role of this CD4^+^ population (Th1) when acting as collaborators to activate CD8^+^ cells. We do not discard a possible mechanism of cross-presentation with CD8^+^ cells recognizing peptides of TSA-1 and Tc24 trough HLA class II molecules. Overall, our findings suggest that peptides from TSA-1 and Tc24 proteins could have different HLA supertype restriction, highlighting the advantage of a vaccine composed of both proteins, which should thus be able to be effective over a wide range of individuals and HLA haplotypes. More studies using peptides derived from TSA-1 and Tc24 are needed to assay peptide restriction to HLA molecules.

In conclusion, we showed that TSA-1 and Tc24 antigens prime the immune system during natural *T*. *cruzi* infection, and induce a long lasting humoral and cellular immune response that can be recalled *in vitro* after at least 10 years of chronic infection. These findings support the immunogenicity of both TSA-1 and Tc24 as potential vaccine candidates in humans. The use of these antigens as a therapeutic vaccine, alone or in combination with drug therapy may help control the development of chronic cardiac disease caused by *T*. *cruzi*. These results represent an important step towards the initiation of pre-clinical trials of such vaccine in non-human primates and future clinical trials.

## Supporting information

S1 Fig Flow cytometry-gating strategy. Brief graphical description of the general procedure used to identify populations of T cells (CD3^+^, CD3^+^CD4^+^ and CD3^+^CD8^+^), cytokine production and phenotyping of memory T cells subpopulations using flow cytometry.
(TIF)

S2 Fig Specific proliferative response t*o* control molecules and antigens. PBMCs from the indicated donors were stimulated in vitro with ConA (A), as well as with BCG (B) and TcSA (C), and analyzed by flow cytometry.
(TIF)

S3 Fig Memory T cells phenotyping following control stimulations. PBMC were stimulated with ConA (A) and TcSA (B), and memory T cells were phenotyped by flow cytometry.
(TIF)

S4 Fig TSA-1 antigen-specific CD4^+^ memory cells phenotyping. (TIF)

S5 Fig Cytokine-producing T cells following control stimulations. PBMC were stimulated with ConA (A) and TcSA (B and C), and cytokine producing CD4^+^ and CD8^+^ T cells were identified by flow cytometry.
(TIF)

S1 Table Six/four digits HLA-A and HLA-B alleles found in Chagasic patients and seronegative individuals and their respective supertypes. (TIF)

S2 Table HLA allele frequencies in some Mexican populations. (TIF)
