# Supplementary figures and images for: Trypanosoma cruzi vaccine candidate antigens Tc24 and TSA-1 recall memory immune response associated with HLA-A and -B supertypes in Chagasic chronic patients from Mexico
Source: PLoS Negl Trop Dis. 2018 Jan 29;12(1):e0006240. doi: 10.1371/journal.pntd.0006240 (PMC5805372; doi:10.1371/journal.pntd.0006240)

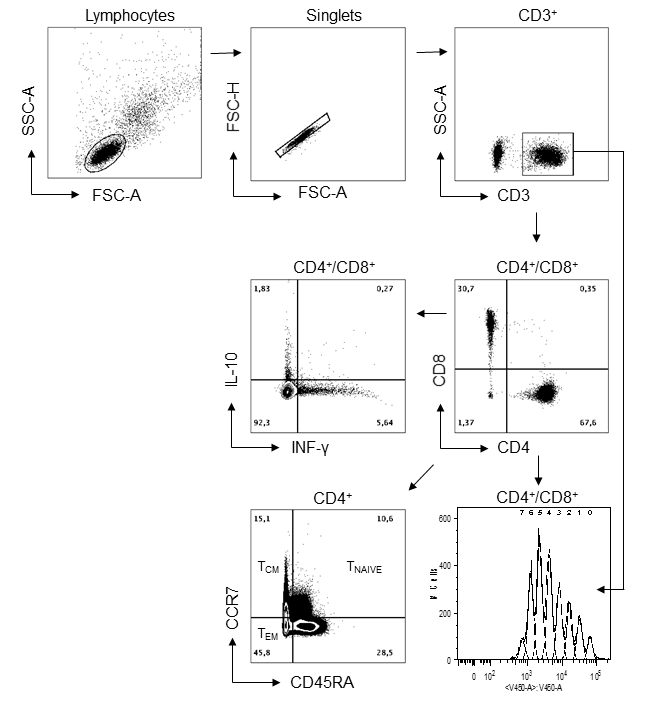

Supplement: S1 Fig — Brief graphical description of the general procedure used to identify populations of T cells (CD3+, CD3+CD4+ and CD3+CD8+), cytokine production and phenotyping of memory T cells subpopulations using flow cytometry. (TIF) [file pntd.0006240.s001.tif]

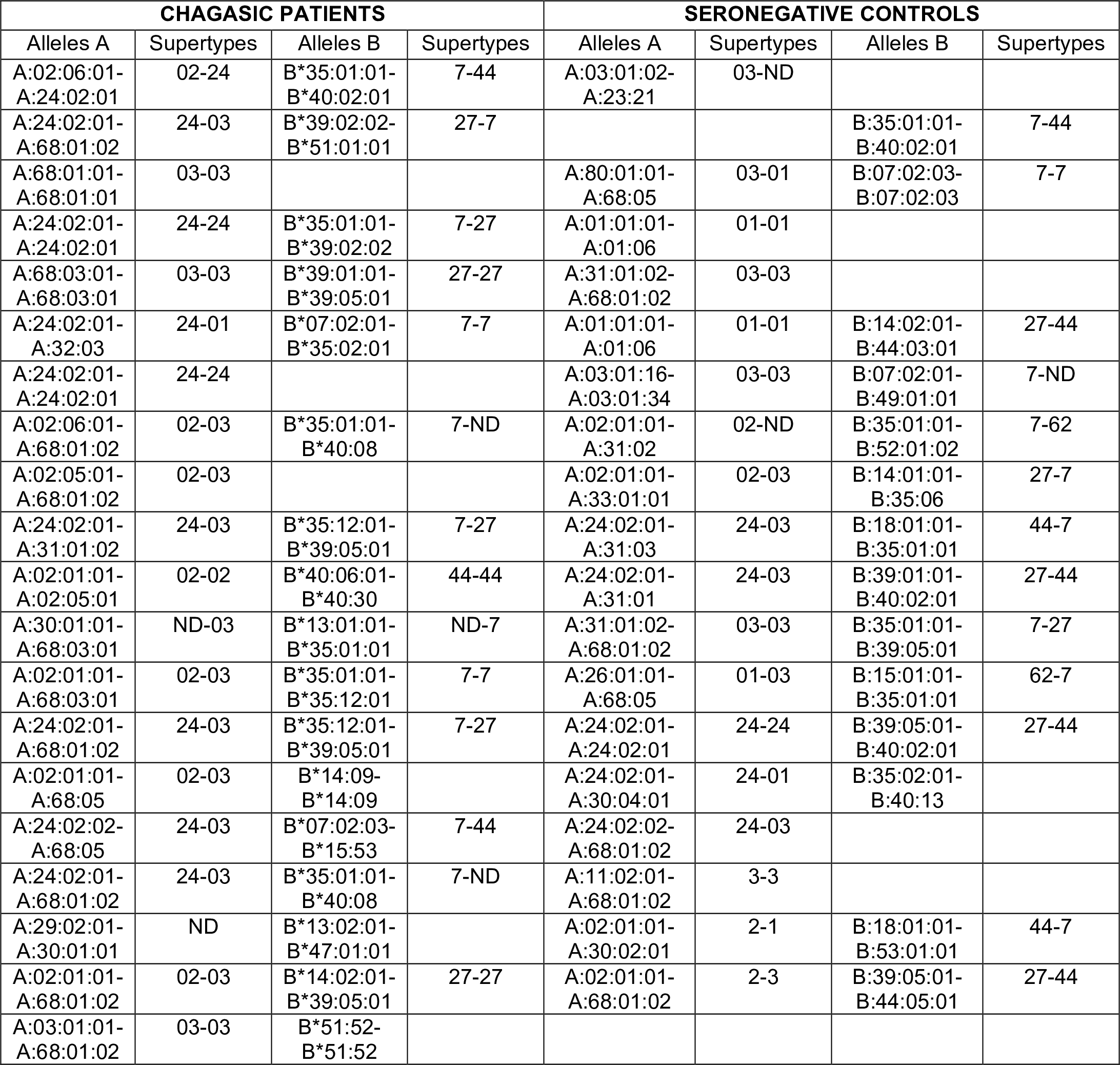

Supplement: S1 Table — (TIF) [file pntd.0006240.s006.tif]

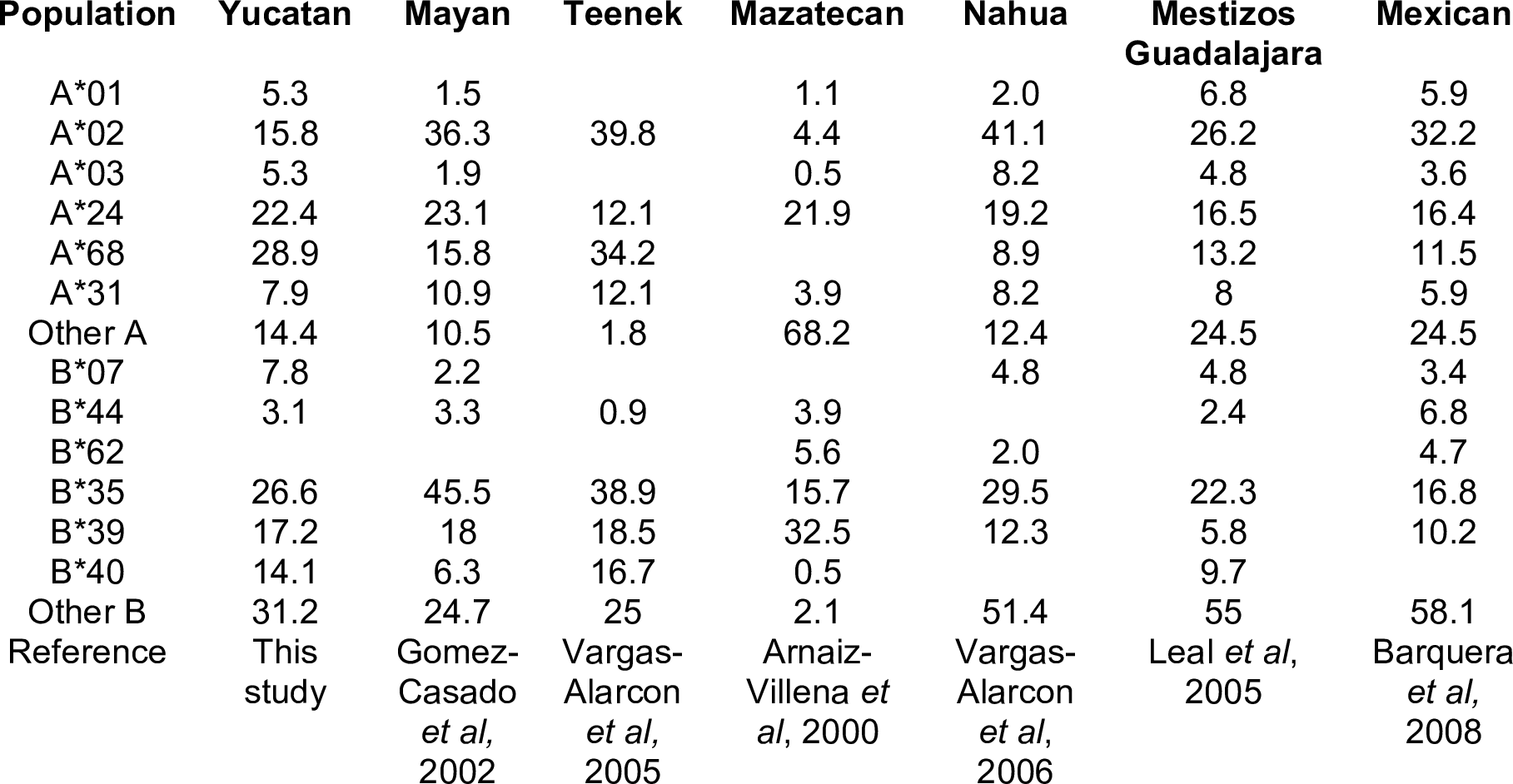

Supplement: S2 Table — (TIF) [file pntd.0006240.s007.tif]
